# Metabolomic Alteration in the Plasma of Wild Rodents Environmentally Exposed to Lead: A Preliminary Study

**DOI:** 10.3390/ijerph19010541

**Published:** 2022-01-04

**Authors:** Hokuto Nakata, Akifumi Eguchi, Shouta M. M. Nakayama, John Yabe, Kaampwe Muzandu, Yoshinori Ikenaka, Chisato Mori, Mayumi Ishizuka

**Affiliations:** 1 Laboratory of Toxicology, Department of Environmental Veterinary Sciences, Faculty of Veterinary Medicine, Hokkaido University, Kita 18 Nishi 9, Kita-ku, Sapporo 060-0818, Japan; hokuto.nakata@vetmed.hokudai.ac.jp (H.N.); y_ikenaka@vetmed.hokudai.ac.jp (Y.I.); 2 Center for Preventive Medical Sciences, Chiba University, Inage-ku Yayoi-cho 1-33, Chiba 263-8522, Japan; a_eguchi@chiba-u.jp (A.E.); cmori@faculty.chiba-u.jp (C.M.); 3 School of Veterinary Medicine, The University of Zambia, P.O. Box 32379, Lusaka 10101, Zambia; mjyabe@yahoo.co.uk (J.Y.); kmuzandu@yahoo.com (K.M.); 4 School of Veterinary Medicine, University of Namibia, P/B. 13301, Windhoek 10005, Namibia; 5 Water Research Group, School of Environmental Sciences and Development, North-West University, Private Bag X6001, Potchefstroom 2531, South Africa; 6 Translational Research Unit, Veterinary Teaching Hospital, Faculty of Veterinary Medicine, Hokkaido University, Sapporo 060-0818, Japan; 7 One Health Research Center, Hokkaido University, Sapporo 060-0818, Japan; 8 Department of Bioenvironmental Medicine, Graduate School of Medicine, Chiba University, Chuo-ku Inohana 1-8-1, Chiba 260-8670, Japan

**Keywords:** lead, metabolomics, wild rodent, mining, biomarker, biological pathway, lasso regression model, random forest model

## Abstract

Lead poisoning is often considered a traditional disease; however, the specific mechanism of toxicity remains unclear. The study of Pb-induced alterations in cellular metabolic pathways is important to understand the biological response and disorders associated with environmental exposure to lead. Metabolomics studies have recently been paid considerable attention to understand in detail the biological response to lead exposure and the associated toxicity mechanisms. In the present study, wild rodents collected from an area contaminated with lead (N = 18) and a control area (N = 10) were investigated. This was the first ever experimental metabolomic study of wildlife exposed to lead in the field. While the levels of plasma phenylalanine and isoleucine were significantly higher in a lead-contaminated area versus the control area, hydroxybutyric acid was marginally significantly higher in the contaminated area, suggesting the possibility of enhancement of lipid metabolism. In the interregional least-absolute shrinkage and selection operator (lasso) regression model analysis, phenylalanine and isoleucine were identified as possible biomarkers, which is in agreement with the random forest model. In addition, in the random forest model, glutaric acid, glutamine, and hydroxybutyric acid were selected. In agreement with previous studies, enrichment analysis showed alterations in the urea cycle and ATP-binding cassette transporter pathways. Although regional rodent species bias was observed in this study, and the relatively small sample size should be taken into account, the present results are to some extent consistent with those of previous studies on humans and laboratory animals.

## 1. Introduction

Lead (Pb) has been and continues to be widely used in industrial activities, owing to its favorable characteristics including ease of smelting and processing, etc. However, it is well established that Pb is a toxic metal that enters the body through the environment, such as soil, dust, water, and food. Numerous previous studies have reported the toxicity of Pb at cellular, tissue or organ levels, which results in biochemical and physiological disorders [1,2]. Lead poisoning is often considered a traditional disease; however, the specific mechanism of toxicity remains unclear. Moreover, Pb poisoning symptoms are non-specific and latent at low exposure levels, without clear clinical signs. Neurodevelopmental disorder is one of the common latent effects of Pb poisoning. Concerned about the potential health effects of Pb at low levels, the Center for Disease Control and Prevention (CDC) has lowered the reference level for blood lead levels in children to 3.5 µg/dL in 2021 [3]. Lead poisoning is considered to have a potentially significant impact on human health in modern society, and careful consideration and regulation is required for industrial use.

The study of Pb-induced alterations in cellular metabolic pathways is important to further understand the biological response and disorders associated with environmental exposure to Pb [4]. Metabolomics represents the complete set of metabolites in a cell, tissue, organ, or whole body. In recent years, it has been attracting more attention as a powerful tool for exploring the alteration of small molecules. Metabolomics is also applied to toxicology through the concept of “toxicometabolomics” [5,6]. Metabolome analysis provides an instantaneous snapshot of the physiology and alterations of chemical processes involving metabolites, small molecule substrates, intermediates, and products of metabolism in response to exposure to environmental contaminants (e.g., metals) [7,8]. For Pb, some metabolomics studies have been conducted on humans using the serum of smelter workers [9], urine of a population living near a Pb-containing battery recycling plant [10], plasma of car commuters [11], and urine from children and elderly individuals living in an industrial coking area [12]. Other studies investigated the metabolic alteration in laboratory rat serum [13] and laboratory mouse feces [14]. However, there is no previous report examining terrestrial field animals environmentally exposed to Pb. As such, there is limited knowledge regarding the relationship between exposure to Pb and metabolomic change. Therefore, further investigation using the metabolomics approach is warranted to characterize the responses of organisms following exposure to Pb.

The town of Kabwe is the capital of the central province of the Republic of Zambia with a long history of Pb and zinc mining, which operated for nearly a century until its closure in 1994. Because of inadequate environmental pollution control during the period of mining activity, high levels of Pb have been reported in the environment [15], wild animals [16,17] including wild rodents [18,19], and humans [20,21]. In our recent study of blood samples from the Kabwe population, we observed an elevation in the levels of Pb in the blood, which was accompanied by alteration of some toxicological parameters [20]. Given this evidence, we considered Kabwe town as a good model area polluted with Pb for our study. The objective of this investigation was to conduct metabolomics research in wild animals by comparing the levels of exposure to Pb.

We aimed to elucidate the metabolic signatures in the affected biological systems induced by exposure to Pb. For this purpose, we selected the well-studied terrestrial animal model of wild rodents. This was the first study investigating the effect of exposure to Pb on metabolomics using field animals.

## 2. Materials and Methods

### 2.1. Rodent Sample Collection

The sampling was performed with permission and strict adherence with the guidelines from the Zambian Ministry of Fisheries and Livestock, as well as the Faculty of Veterinary Medicine, Hokkaido University, Sapporo, Japan (approval number: Vet-17010). In September 2020, wild rodents were captured in two areas of Kabwe District including Mutwe Wansofu (N = 18) and Kang’omba (N = 10). The Mutwe Wansofu was considered polluted because the sample collection point of Mutwe Wansofu is located immediately south to the mine site (distance: ≤250 m). The Kang’omba area is located approximately 5 km from the mine site; therefore, it was included as the control site (Supplementary Figure S1). Box-type cage traps with food as bait were used to capture live wild rodents. Traps were set up in residential areas during the day and retrieved the following morning. Since rodents are generally nocturnal, this method of sampling was efficient. The captured rodents were euthanized by inhalation of carbon dioxide gas and dissected to collect the liver, kidneys, and eyes, as well as whole blood from the postcava, using a heparinized syringe and needle. Prior to the dissection, body weight and sex were determined. A portion of whole blood samples was immediately centrifuged for 10 min at 1500× *g*. Subsequently, the supernatants of plasma samples, as well as the liver, kidney, and remaining whole blood samples were stored at −20 °C. The samples were transported to Japan in cooler boxes for laboratory analysis in the Laboratory of Toxicology, Faculty of Veterinary Medicine, Hokkaido University (Sapporo, Japan), after an international sanitary certificate was obtained from the Zambian Ministry of Fisheries and Livestock (No. 20599).

### 2.2. Identification of Rodent Species

Genomic DNA was extracted from liver samples using the Wizard^®^ Genomic DNA purification kit (Promega Corp., Madison, WI, USA) for species identification through the cytochrome b (*cyt-b,* 762 bp) gene with accession number JX887164.1. The identification method was slightly modified from the original method [22]. The used primers were as follows: forward primer (5′-GGTGAAGGCTTCAACGCCAACCCTA-3′) and reverse primer (5′-TAGAATATCAGCTTTGGGTGTTGATGG-3′). The thermal cycler LifeECO (Nippon Genetics Co., Ltd., Tokyo, Japan) was used for polymerase chain reaction (PCR) amplification. The reaction included a 2 min initial denaturing step at 94 °C followed by 35 cycles at 94 °C for 30 s, 57 °C for 30 s, and 72 °C for 60 s, and a final extension step at 72 °C for 5 min. The volume of PCR mix was 20 µL containing 50 ng of genomic DNA, 10 µL of EmeraldAmp^®^ PCR master mix (Takara Bio Inc., Shiga, Japan) and 0.2 µM of each primer. The purification of the PCR products was performed using the QIAquick^®^ PCR purification kit (Qiagen, Hilden, Germany) according to the instructions provided by the manufacturer. Sequencing was performed by Fasmac Co., Ltd. (Kanagawa, Japan). The rodent species were identified from the obtained sequences through blast analysis using the National Center for Biotechnology Information website.

### 2.3. Estimation of the Age of Rodents

The ages of *Rattus rattus* (*R. rattus*) and *Mastomys natalensis* (*M. natalensis*) were estimated based on the weight of the desiccated eye lens using a slightly modified version of the methods originally described by Tanikawa [23] and Fichet-Calvet et al. [24]. After the fixation of eyes in 10% neutral buffered formaldehyde solution at room temperature for 4 weeks, the tissues around the lens were removed. The lenses were washed with distilled water and dried at 60 °C for 3 days in an oven before weighing. Age estimation was conducted using the following formulae [23,24]:

*y* = 10^(1.02 + 0.023 *x*) (for *R. rattus*)

*y = e*^([10.4608 *+ x*]/4.35076) (for *M. natalensis*)

where *y* is the age (in days), and *x* is the total weight of both lenses (in milligrams).

### 2.4. Extraction and Analysis of Pb

All laboratory materials and instruments used in the heavy metal analysis were washed with 2% nitric acid (HNO_3_) and rinsed at least twice with distilled water. We confirmed that there was no metal contamination through the analytical procedures using the regent (digestion) blank measurement. Whole blood, liver, and kidney samples were digested for metal analysis using a method described in a previous study [17,25] with minor modifications. In brief, approximately 300 mg of each tissue sample was dried for 48 h in an oven at 50 °C. Whole blood (100 µL) and the dried tissue samples were digested with 5 mL of nitric acid (atomic absorption spectrometry grade, 60%; Kanto Chemical Corp., Tokyo, Japan) diluted to 30% and 1 mL of 30% hydrogen peroxide (Cica reagent: 30%; Kanto Chemical Corp., Tokyo, Japan). This digestion process was performed using a microwave digestion system (Speed Wave MWS-2; Berghof, Eningen, Germany) according to the instructions provided by the manufacturer. After cooling, extracted solutions were transferred into 15 mL plastic tubes and diluted to a final volume of 10 mL with bi-distilled and deionized water (Milli-Q; Millipore, Bedford, MA, USA).

The concentration of Pb in the extracted solutions was determined by using an inductively coupled plasma–mass spectrometer (ICP-MS 7700 series; Agilent Technologies Inc., Tokyo, Japan), following the procedure previously described by Nakata et al. [25]. Analytical quality control (QC) was conducted using the certified reference materials of DOLT-4 (dogfish liver; National Research Council of Canada) and Seronorm^TM^ Trace Elements Whole Blood L-2 (Sero, Billingstad, Norway). Replicate analysis of these reference materials showed good accuracy (relative standard deviation was <3%) and recoveries (95–105%). The instrument detection limit was 0.001 µg/L.

### 2.5. Metabolome Analysis

The imported plasma samples were transported to the Center for Preventive Medical Sciences, Chiba University (Chiba, Japan) for the metabolome analysis. A solution containing methanol, ultrapure water, and chloroform (5:2:2 in volume ratio) was prepared. Methanol (99.7+%, for liquid chromatography) and chloroform (99.7+%, for high-performance liquid chromatography) were purchased from Wako Pure Chemical Industries (Osaka, Japan), while ultrapure water was obtained using an RDF280NC system (Advantec, Dublin, CA, USA). Each plasma sample (10 µL) was mixed to prepare QC. Fifty microliters of plasma samples and QC were mixed with 250 µL of the solution and then mixed with 10 µL of internal standard solution containing 0.2 mg/mL of adonitol (Sigma–Aldrich, Tokyo, Japan) in methanol and centrifuged (14,000 rpm, 4 °C, 5 min). Subsequently, 200 µL of Milli-Q water was added to 250 µL of supernatant. The samples were centrifuged again (14,000 rpm, 4 °C, 5 min). To remove ethanol, the supernatants were placed in a centrifugal evaporator (CVE-2100; Tokyo Rikakikai Co, Ltd., Tokyo, Japan) equipped with a vacuum system (V-700; Shibata Scientific Technology Ltd., Saitama, Japan) for 2 h. Next, the residues were placed in a pre-cooled (at −80 °C) glass tube for overnight freeze-drying using FDS-1000 (Tokyo Rikakikai Co., Ltd.).

Methoxyamine hydrochloride (20 mg/mL, 80 µL) (GL Sciences Inc., Tokyo, Japan), which was dissolved in pyridine (Wako Pure Chemical Industries) immediately prior to use, was added to the freeze-dried samples for reconstitution. Subsequently, the reconstituted samples were incubated (1200 rpm, 30 °C, 90 min) for the first derivatization of methoxylation. For the second derivatization of trimethylsilylation, 40 µL of N-methyl-N-trimethylsilyl-trifluoroacetamide was added and centrifuged (14,000 rpm, room temperature, 5 min) after incubation (1200 rpm, 37 °C, 30 min). Finally, the supernatant was transferred to the gas chromatography–mass spectrometry (GC–MS) vial. Within 24 h after the completion of derivatization, the metabolome analysis was demonstrated using JMS-Q 1500GC (Jeol Ltd., Tokyo, Japan) quadrupole mass spectrometer equipped with an Agilent 7890B gas chromatograph and a 7693 autosampler (Agilent Technologies Inc., Tokyo, Japan). An Rxi^®^-5Sil MS column (30 m length × 0.25 mm I.D. × 0.25 µm film thickness; Shimadzu GLC Ltd., Tokyo, Japan) was used for GC separation. For quality assurance, QC samples were analyzed at the beginning of the sequence and every seven samples, as well as a pyridine blank at the beginning. Additionally, a hydrocarbon mixture standard (C_9_–C_40_, all even in n-Hexane) (GL Science Inc., Tokyo, Japan) was analyzed at the end of the sequence for the correction of retention time. Peak selection and annotation were performed using MS-DIAL version 4.36 with GC–MS DB-Public-KovatsRI-VS3.msp library [26]. Peak heights were normalized to those of the internal standard and a QC-sample-based, robust, locally estimated scatterplot smoothing.

### 2.6. Statistical Analysis

The *t* test, Mann–Whitney U test, and Shapiro–Wilk test were performed using the IBM SPSS Statistics version 26.0 software (IBM Corporation, Armonk, NY, USA) to examine the differences in animal characteristics and levels of Pb between groups, as well as the data normality. The Shapiro–Wilk test revealed lack of data normality for the body weight, estimated age, and levels of Pb in blood, liver, and kidney. Thus, the data were log-transformed and subsequently confirmed for normal distribution using the Shapiro–Wilk test, except for body weight. For this reason, the differences between groups for body weight were examined using the Mann–Whitney U test without log-transformation, while the *t* test was used for the other parameters after log-transformation. A *p* value of <0.05 denoted statistically significant differences.

The R packages in R.4.0.5 [27] were used for further statistical analysis. We conducted principal component analysis (PCA) and constructed a correlation matrix by using the data of the detected metabolites to determine metabolomic profiles. The Mann–Whitney *U* test and Shapiro–Wilk test were used to investigate the differences in metabolites across sampling sites. Metabolomes were modeled through “least absolute shrinkage and selection operator” (lasso) [28] using the R package glmnet [29] and random forests [30] using the R package ranger [31] involving metabolites detected in ≥50% of samples and with a coefficient of variation <40% (25 metabolites). For the investigation of changes induced by Pb exposure, animals collected from the polluted site (Mutwe Wansofu) were compared with those collected from the control site (Kang’omba) by using logistic regression with the L1 regularization lasso model [28] in the R package glmnet [29] and the random-forests model [30] in the R package ranger [31]. Logistic regression with L1 regularization (lasso) and random forests were used to predict the sampling location from metabolites and age data. The number of variables sampled randomly as candidates in each split of the lasso and random forest model was optimized using the R package caret [32]. In all cases, the training sets consisted of 60% of samples from each site, while the remaining 40% were used as external test sets, which generated random numbers in the R selected. Lasso logistic regression and random-forest models were also assessed through repeated five-fold cross-validation (10 replicates) using multivariate receiver operating characteristics (ROC) and areas under the ROC curves (AUC) as measures of robustness. Likewise, R-square (R^2^) was used to further assess the lasso linear regression models. Parameters that optimized the R^2^ were determined numerically. Metabolites retained with variable importance of >0 in the linear and logistic regression with L1 regularization models were considered potential biomarkers. Furthermore, random-forest analysis for regression analysis was performed to distinguish the sampling location based on the metabolome profile and levels of Pb in blood, but the levels of Pb in the liver and kidney, species, and sex were excluded. Metabolites with a *p* value of <0.05, calculated using the permutation variable importance method [33] and showing significant differences between regions by Mann–Whitney U test and also identified by the lasso logistic model, were identified as potential biomarkers. Enrichment analysis of metabolic pathways was carried out using MBROLE2 [34] to distinguish sampling location; *p* values were adjusted by controlling the false discovery rate (FDR) [35]. A stringent FDR (q value) of <0.2 was used to identify metabolic pathways by using the Kyoto Encyclopedia of Genes and Genomes [36], the Human Metabolome Database [37], and the BioCyc Database [38] identifier to annotate five candidates including two potential biomarkers and three other metabolites with a *p* value of <0.05 in random forest analysis (hydroxybutyric acid, glutaric acid and glutamine).

## 3. Results

### 3.1. Characteristics and Pb Levels of Animals

Data on the identified species, estimated age, body weight, as well as Pb levels in the blood, liver, and kidney, are summarized according to species and area in Table 1. In this study, 18 rodents (17 *R. rattus* and one *M. natalensis*) collected from Mutwe Wansofu and 10 rodents (two *R. rattus*, one *R. tanezumi*, and seven *M. natalensis*) collected from Kang’omba were used for further analysis; notably, there was a species bias across the areas. The Mann–Whitney U test demonstrated the significantly greater body weight of rodents collected from the polluted site versus the control site. In contrast, the *t* test showed that the estimated age of animals was significantly higher in the control site versus the polluted site. The measured levels of Pb in blood, liver, and kidney showed significant elevation in the samples collected from the polluted site, confirming the pollution status.

### 3.2. Metabolome Profiles and Pathway Enrichment Analysis

**Figure 1:**
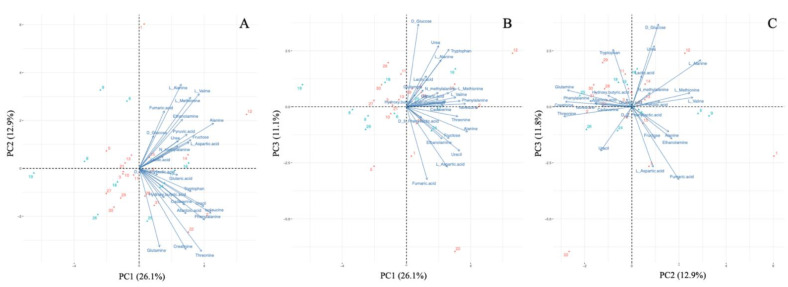
Principal component analysis of metabolites. (**A**) First principal component (PC1) versus second principal component (PC2). (**B**) PC1 versus third principal component (PC3). (**C**) PC2 versus PC3. Red dots indicate animals from the polluted site (Mutwe Wansofu), and blue dots indicate animals from the control site (Kang’omba).

The GC–MS analysis detected a total of 25 target metabolites in >50% of plasma samples (<40% coefficient of variation). The clustering pattern of samples was visualized through PCA (Figure 1). The PCA results demonstrated that the first (PC1), second (PC2), and third (PC3) principal components respectively accounted for 26.1%, 12.9%, and 11.1% of the variation. There were no obvious regional clusters. All of the metabolites positively determined PC1, while they were vectored in diverse directions in Figure 1C, which is a combination of PC2 and PC3. There was no clear trend observed; however, for instance, isoleucine and phenylalanine were positively related to PC1 and negatively related to PC2, while D-glucose and lactic acid were positively associated with PC1, PC2, and PC3.

**Figure 2:**
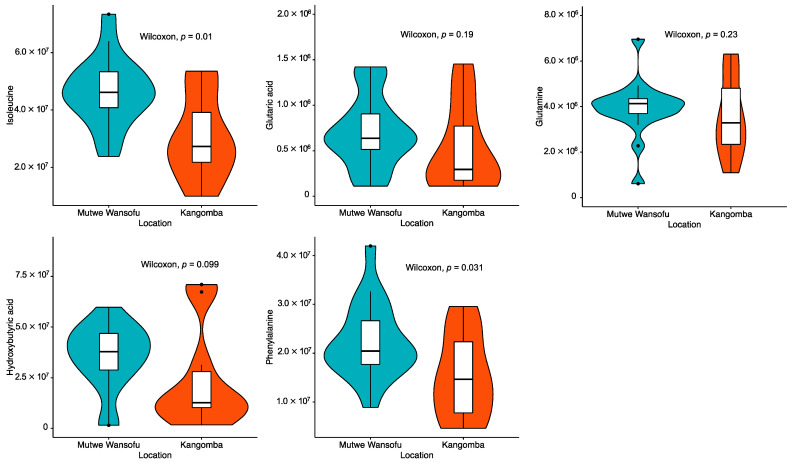
Comparison of the measured concentration (peak area) of plasma metabolites according to area.

**Figure 3:**
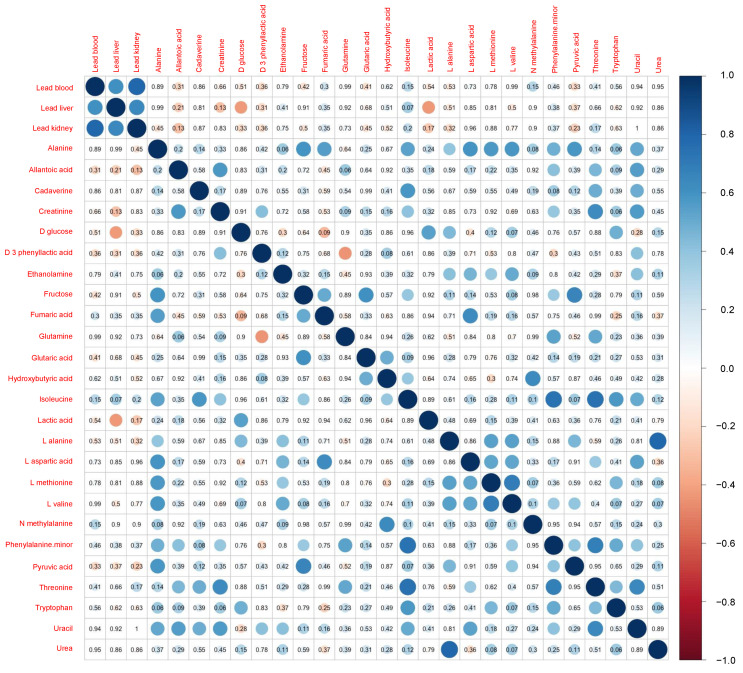
Correlation matrix of the levels of lead in tissue and concentration of metabolites in plasma. The number in each cell indicates the *p* value, and the colored circle indicates the correlation coefficient.

Figure 2 shows a comparison of the results of the metabolomics analysis according to the area. The Mann–Whitney U test revealed significantly higher levels of plasma isoleucine (*p* = 0.011) and phenylalanine (*p* = 0.031) in the polluted site versus the control site. A marginally significant increase in the levels of hydroxybutyric acid (*p* = 0.099) in the polluted site was also exhibited. The correlation matrix for the rodent characteristics and concentrations of Pb is shown in Figure 3. Apart from the correlation across the concentrations of Pb in blood, liver, and kidney, only two metabolites (i.e., plasma D-glucose and lactic acid) showed a significant negative correlation with the levels of Pb in the liver. Moreover, a marginally significant positive correlation was detected between the concentration of Pb in the liver and the levels of isoleucine in plasma (*p* = 0.07).

**Figure 4:**
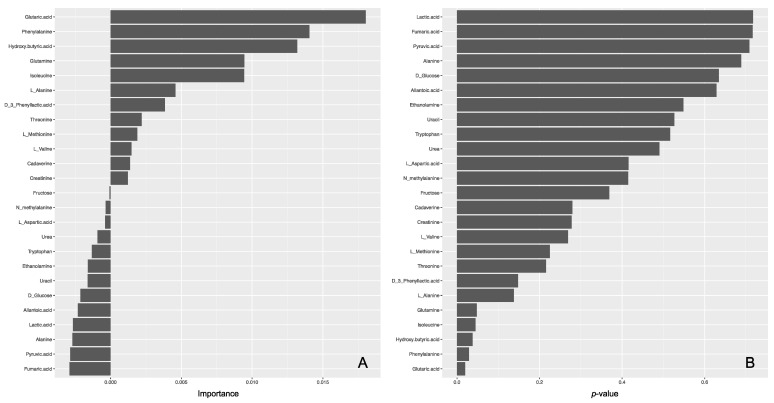
Importance (**A**) and *p* values (**B**) of metabolites in the random forest model.

The lasso logistic regression model was moderately adequate on the basis of its AUC values against a training data set in the five-fold cross-validation and an external test set (0.8 and 0.714, respectively). As a result, only two metabolites (isoleucine and phenylalanine) were identified as important variables in the lasso for the regression model (Supplementary Figure S2). In the random-forest model, the AUC was moderately good on the basis of its AUCs against a training data set after optimization by five-fold cross validation and an external test set (0.727 and 0.714, respectively). Of the 25 used variables, five variables (i.e., phenylalanine, isoleucine, hydroxybutyric acid, glutaric acid, and glutamine) showed a *p* value of <0.05 (Figure 4). Of these five metabolites, phenylalanine and isoleucine showed significant differences between regions by Mann–Whitney U test and were also identified by the lasso logistic model, suggesting that they would be potential biomarkers.

Analysis using MBROLE2 at FDRs of <0.2 and at least two compounds per metabolic pathway revealed that the candidates were associated with various pathways (i.e., urea cycle; superpathway of phenylalanine, tyrosine, and tryptophan biosynthesis; phenylalanine degradation IV; tRNA charging; indole-3-acetyl-amide conjugate biosynthesis; superpathway of indole-3-acetate conjugate biosynthesis; aminoacyl-tRNA biosynthesis; and ATP-binding cassette (ABC) transporters) for both the lasso logistic regression model and random forest model. In the same analysis, the jasmonoyl-amino acid conjugates biosynthesis I; jasmonoyl-amino acid conjugates biosynthesis II; biosynthesis of alkaloids derived from ornithine, lysine and nicotinic acid; glucosinolate biosynthesis; and tropane, piperidine, and pyridine alkaloid biosynthesis pathways were recorded for the random forest model only (Supplementary Table S1).

## 4. Discussion

In this study, we captured wild rodents from a Pb-contaminated site and a control site to verify the metabolomic alterations induced by environmental exposure to Pb. This was the first study to evaluate the relationship between environmental exposure to Pb and alteration of metabolomics in terrestrial wild animals. Eighteen and 10 rodents from the polluted and control sites, respectively, were used for metal and metabolomics analysis. An animal species bias was observed in the two areas. This is one of the limitations of this study; however, due to the nature of field research, it is often difficult to avoid. Most of the animals in the contaminated and control sites were *R. rattus* and *M. natalensis*, respectively. Mastomys is the most widespread kind of rodent in the African continent [39], diverging 11.3 ± 0.5 My from rats and 10.2 ± 0.6 My from mice [40,41]. However, there is limited knowledge regarding the biological characteristics of Mastomys. In contrast, *R. rattus* is well investigated and widely used as a sentinel animal in environmental research. Anatomical and morphological differences between these two species of rodents have been reported [42,43]. Nevertheless, the differences between these animal species in metabolomics are completely unknown. A report on establishing a breeding colony of *M. natalensis* was recently published [44]. Therefore, it is expected that *M. natalensis* will be investigated further in the near future; however, the currently available data are limited.

For the above reasons, in the data analysis, we treated all animals as a single group in each site. Although there was a significant difference in body weight and estimated age between the two sites, it is unlikely that these had a significant effect on the patterns of Pb accumulation in tissues, according to the findings of previous studies [15,19]. The significantly elevated levels of Pb detected in the blood, liver, and kidney of animals collected from the site near the mine were consistent with those reported in previous studies in wild rodent animals [18,19] and confirmed the pollution in this area.

Our study revealed a significant increase in isoleucine and phenylalanine in the plasma of rodents collected from the Pb-contaminated site. In the PCA, these two metabolites exhibited almost identical vectors in the same direction. The correlation matrix showed a marginally significant association between the levels of Pb and isoleucine in the liver, in agreement with the significant elevation observed in the polluted site. However, the correlation matrix did not demonstrate a significant correlation between the levels of Pb and phenylalanine. Similarly, there was a marginally significant difference in the concentration of hydroxybutyric acid between areas, but it was not correlated with the concentration of Pb. Hydroxybutyric acid is a major ketone body synthesized from acetyl-coenzyme A in the liver via the fatty acid metabolism. When the energy supply from carbohydrates is insufficient, the liver compensates by promoting the degradation of lipids to produce hydroxybutyric acid. In turn, hydroxybutyric acid is used by the body as an alternative energy source. Previous studies suggested that exposure to Pb induces disturbance of the lipid metabolism in smelter workers exposed to arsenic, cadmium, and Pb [9], as well as in adult male zebrafish (*Danio rerio*) [45]. Additionally, Gao et al. [14] showed that gut-microbiome-community structures and bile acid homeostasis were affected in C57BL/6 mice exposed to Pb and suggested that these changes affected energy metabolism. Conversely, there are multiple pathways for the synthesis and metabolism of acetyl-coenzyme A. It is conceivable that an increase in hydroxybutyrate production by metabolism of acetyl-coenzyme A may have occurred as a secondary effect due to changes occurring in other pathways. It is difficult to discuss this point in detail based on the results of this study and the previous studies available at this moment, but it is a point that should be the focus of future research. Additionally, since our results did not show a significant association between the levels of Pb and hydroxybutyric acid, other two possibilities should be considered. One such possibility is differences in the nutritional status due to regional differences in dietary habits or available food resources. Insufficiency of carbohydrates in animals that inhabited the Pb-contaminated area is implied by the negative association of D-glucose and lactic acid, which are the substrate and metabolite of glycolysis, respectively, with the levels of Pb noted in the correlation matrix. Another possible reason is differences in energy metabolism between the *R. rattus* and *M. natalensis* species. Our results implied that *M. natalensis* rodents could be more likely to use fatty acids as an energy source than carbohydrates. However, although species differences in lipid metabolism at the class level have been determined [46], there is limited knowledge on species differences among rodents. In this study, the animals in the contaminated area had lower carbohydrate metabolism and higher lipid metabolism compared with those in the control area. Nevertheless, it is unclear whether this is caused by endogenous reasons (e.g., differences in animal species) or exogenous reasons (e.g., differences in nutrient sources).

The significantly higher levels of phenylalanine detected in the plasma of animals collected from the Pb-contaminated areas in this study are consistent with those previously reported that suggested the disturbance of amino acid metabolism in smelter workers [9]. Isoleucine, another essential amino acid, was identified along with phenylalanine by the lasso linear regression model in our study. Currently, there is insufficient knowledge regarding the changes in isoleucine caused by exposure to Pb; thus, the interpretation of this result is difficult.

A random-forest model was also applied to distinct areas. The model identified some amino acids, including those (phenylalanine and isoleucine) highlighted by the aforementioned analyses. The identification of hydroxybutyric acid was supportive of the possible effect of Pb exposure on lipid metabolism, as suggested by previous reports [9,14,45]; however, thus far, there are no reports concerning glutaric acid and glutamine. The same compound was shown as a candidate biomarker in metabolomics studies involving different animal species and sample types. Nevertheless, the selected potential biomarkers in our study did not overlap with the molecules reported in a previous study that examined adult male Wistar rats experimentally exposed to Pb [13]. Of note, the analytical methods used for metabolome analysis in studies differed (i.e., GC–MS in this study and liquid chromatography–MS in previous studies). Such differences in analytical methods are generally recognized as a challenge in the field of metabolomics research and should be taken into account.

**Figure 5:**
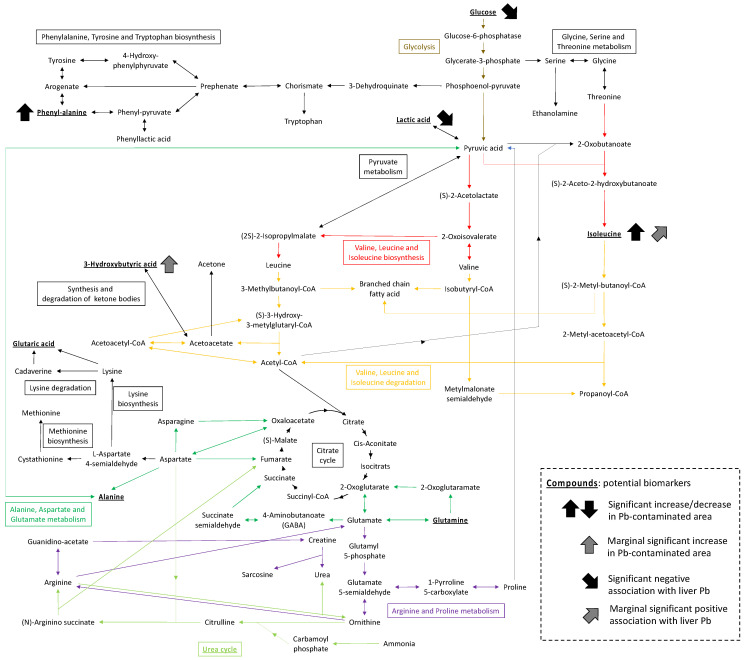
Suggested biomarkers (bold and underline) and metabolic pathways identified in rodents in this study.

Enrichment analysis of altered metabolic pathways in plasma highlighted several pathways related to the degradation and biosynthesis of amino acids, as well as some chemical compounds with an FDR of <0.2. Figure 5 summarizes the potential biomarkers and pathways identified in our analysis. The ABC transporters and urea cycle were previously detected in a metabolomics analysis of urine collected from a Vietnamese population exposed to Pb from a smelter [10]. Moreover, the results concerning ABC transporters are in line with those obtained in an experimental exposure study involving C57BL/6 mice [14]. Identical pathways were identified for the different species, although the interpretation of observations between species and sample types should be made with caution. This study had some limitations, such as animal species bias and a relatively small sample size due to its nature (i.e., field-based study). However, the present findings contribute to the accumulation of knowledge with regard to metabolomic changes following exposure to Pb and showed consistency with previous data obtained from human and laboratory animal studies.

Under the concept of “Toxicometabolomics”, metabolomic studies in toxicology have become increasingly important in recent years. Conversely, metabolomic knowledge is still insufficient not only for Pb, but also for other metals. Although in vivo studies have generally taken the lead, it is desirable to accumulate knowledge in the field as in this study. In our study, it was difficult to demonstrate the further analysis regarding other metals due to insufficient sample size and sample bias. In the future, it is expected to expand the target metals to other metals and to study the combined effects of multiple metals.

## 5. Conclusions

This was the first metabolomics study of wild rodents environmentally exposed to Pb in the field. The blood, liver, and kidneys of animals captured in the contaminated area accumulated significantly higher concentrations of Pb than those of animals in the control area. Metabolomic analysis of plasma was carried out by GC-MS. The high levels of hydroxybutyric acid detected in the contaminated area suggest an enhanced lipid metabolism in animals; however, the possibility that this observation may be due to species bias or regional dietary differences should be considered. Phenylalanine and isoleucine were identified as possible biomarkers through comparison of regional differences, lasso analysis and random-forest model. In addition to the three compounds mentioned above, the random-forest model also identified glutaric acid and glutamine. Consistent with previous studies, the urea cycle and ABC transporters pathway were identified among the altered metabolic pathways. Although the relatively small sample size and the existence of animal species bias in different areas should be considered as study limitations, the results obtained in this study are extremely important for understanding Pb toxicity and its mechanisms. Importantly, a certain degree of commonality was found between previous studies on Pb-exposed humans and experimental animals and the present study on wild animals.

## Figures and Tables

**Table 1:** Identified species, characteristics, and tissue Pb levels of the target animals.

	Species and Sample Size	Sex (Male, Female)	Body Weight * (g)	Estimated Age ** (day)	Blood Pb ** (µg/dL)	Liver Pb ** (mg/kg, dw)	Kidney Pb *** (mg/kg, dw)
Mutwe Wansofu (polluted site)	Total (*N* = 18)	14, 4	108 ± 44.3	139 ± 107	33.6 ± 25.4	5.15 ± 3.98	14.2 ± 12.2
	*R. rattus* (*N* = 17)	14, 3	113 ± 40.5	190 ± 163	33.1 ± 26.0	4.36 ± 2.24	13.6 ± 12.3
	*M. natalensis* (*N* = 1)	0, 1	25.2	203	42.9	15.8	24.4
Kang’omba (control site)	Total (*N* = 9)	5, 4	49.5 ± 30.1	227 ± 136	5.62 ± 1.86	1.07 ± 0.542	4.26 ± 1.03
	*R. rattus* (*N* = 2)	1, 1	91.3, 107	75.9, 234	5.57, 6.44	0.483, 0.624	2.85, 4.83
	*M. natalensis* (*N* = 7)	4, 3	35.3 ± 11.5	248 ± 142	5.50 ± 2.11	1.22 ± 0.524	4.37 ± 1.01

* p < 0.005, ** *p* < 0.0001, *** *p* < 0.05.

## Data Availability

The datasets generated and analyzed during the current study are available from the corresponding author on reasonable request.

## Supplementary Materials

The following are available online at https://www.mdpi.com/article/10.3390/ijerph19010541/s1, Figure S1: Map of Kabwe town showing a mine dump site and two sampling sites, including Mutwe Wansofu and Kang’omba (modified from Google Earth), Figure S2: Importance of the selected variables in the lasso logistic model. Table S1: Outcomes of enrichment analysis of altered metabolic pathways identified as potential biomarkers in the lasso logistic regression model and random forest model.
