# Supplementary material for: Metabolomic Alteration in the Plasma of Wild Rodents Environmentally Exposed to Lead: A Preliminary Study
Source: Int J Environ Res Public Health. 2022 Jan 4;19(1):541. doi: 10.3390/ijerph19010541 (PMC8744629; doi:10.3390/ijerph19010541)
Supplement: Supplementary file 1 [file ijerph-19-00541-s001.zip › ijerph-1497739-supplementary.pdf]

Supplemental Material

Metabolomic alteration in the plasma of wild rodents environmentally exposed to lead: a preliminary study

Hokuto Nakata, Akifumi Eguchi, Shouta M.M. Nakayama, John Yabe, Kaampwe Muzandu, Yoshinori Ikenaka, Chisato Mori, Mayumi Ishizuka

Table of Contents

Supplementary Figure S1. Map of Kabwe town showing a mine dump site and two sampling sites, including Mutwe Wansofu and Kang'omba (modified from Google Earth).

Supplementary Figure S2. Importance of the selected variables in the lasso logistic model.

Supplementary Table S1. Outcomes of enrichment analysis of altered metabolic pathways identified as potential biomarkers in the lasso logistic regression model and random forest model.

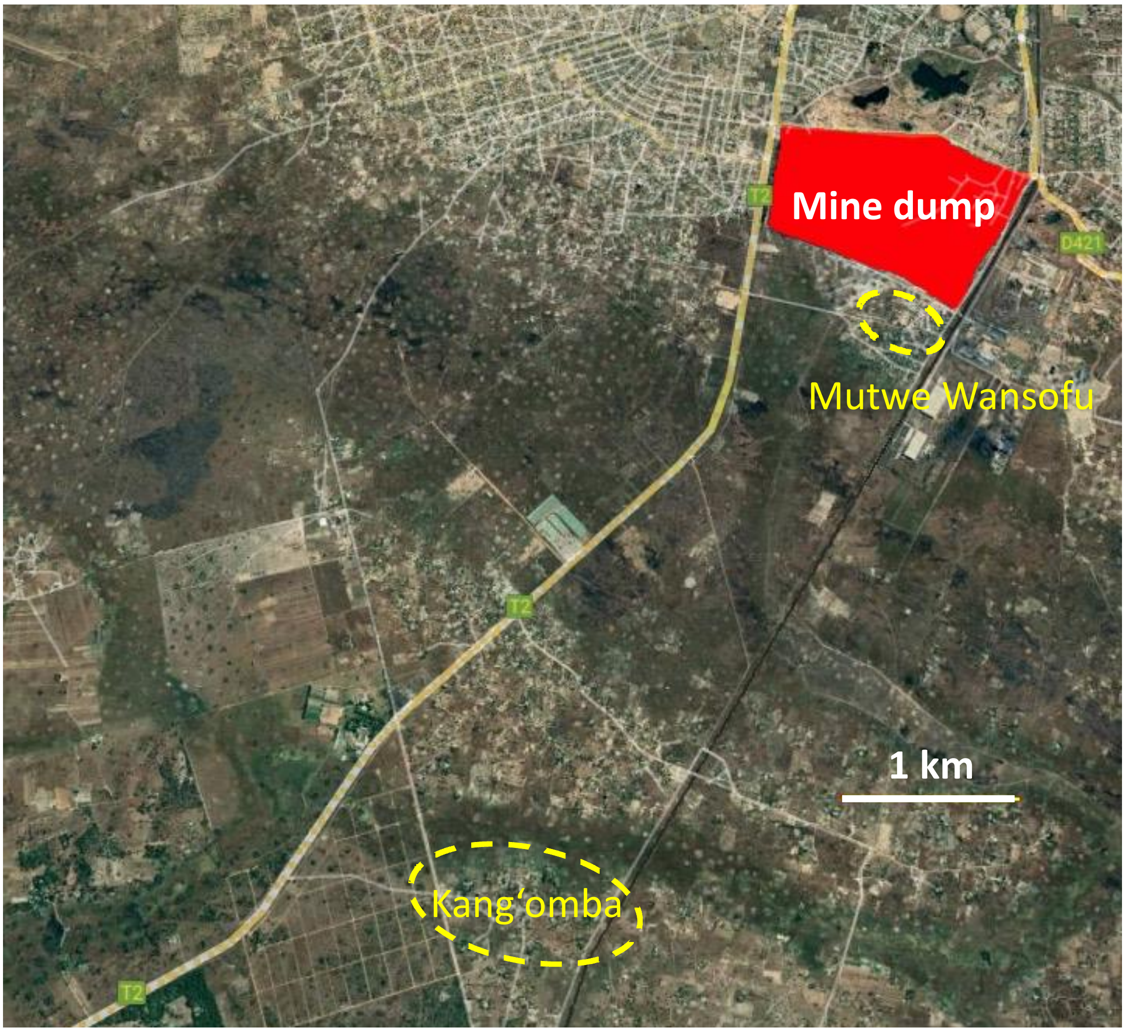

Supplementary Figure S1. Map of Kabwe town showing a mine dump site and two sampling sites, including Mutwe Wansofu and Kang'omba (modified from Google Earth).

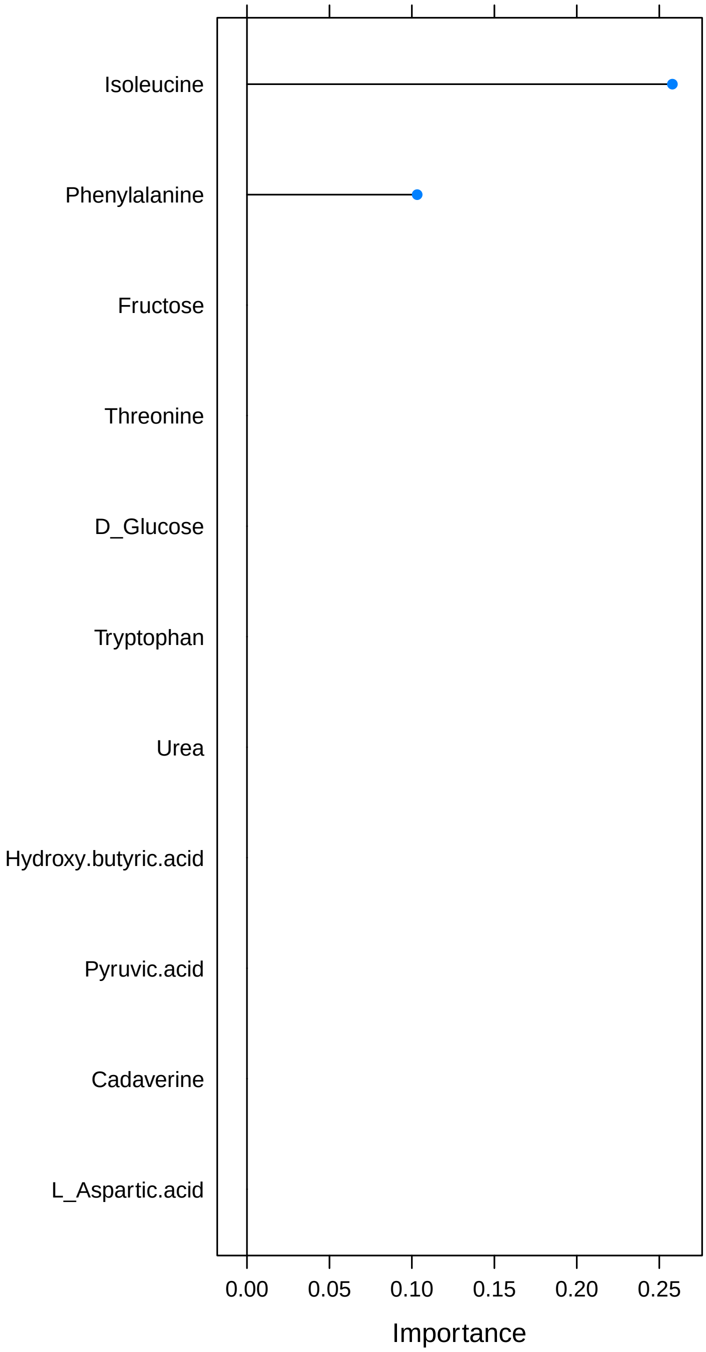

Supplementary Figure S2. Importance of the selected variables in the lasso logistic model.

Abbreviations: lasso, least absolute shrinkage and selection operator

Supplementary Table S1. Outcomes of enrichment analysis of altered metabolic pathways identified as potential biomarkers in the lasso logistic regression model and random forest model.

<table>
<tr><th colspan="8">For lasso logistic regression model</th></tr>
<tr><th>ID Annotation</th><th>Annotation</th><th>Category</th><th>Database</th><th>p-value</th><th>FDR correction</th><th>Submitted IDs</th><th>Matching IDs</th></tr>
<tr><td>SMP00059</td><td>Urea Cycle</td><td>SMPDB Pathway</td><td>ECMDB</td><td>0.0043</td><td>0.0658</td><td>ChEBI:17295|ChEBI:18050</td><td>ECMDB00159|ECMDB00641</td></tr>
<tr><td>SMP00019</td><td>Transcription/Translation</td><td>SMPDB Pathway</td><td>HMDB</td><td>0.0097</td><td>0.0876</td><td>ChEBI:17295|ChEBI:18050</td><td>HMDB00159|HMDB00641</td></tr>
<tr><td>COMPLETE-ARO-PWY-1</td><td>superpathway of phenylalanine, tyrosine, and tryptophan biosynthesis</td><td>Pathways</td><td>BioCyc</td><td>0.0033</td><td>0.1289</td><td>ChEBI:18050|ChEBI:17295</td><td>GLN|PHE</td></tr>
<tr><td>PWY-6318</td><td>phenylalanine degradation IV</td><td>Pathways</td><td>BioCyc</td><td>0.0089</td><td>0.1289</td><td>ChEBI:18050|ChEBI:17295</td><td>GLN|PHE</td></tr>
<tr><td>TRNA-CHARGING-PWY</td><td>tRNA charging</td><td>Pathways</td><td>BioCyc</td><td>0.0422</td><td>0.1289</td><td>ChEBI:18050|ChEBI:17295</td><td>GLN|PHE</td></tr>
<tr><td>PWY-6219</td><td>indole-3-acetyl-amide conjugate biosynthesis</td><td>Pathways</td><td>BioCyc</td><td>0.0041</td><td>0.1289</td><td>ChEBI:18050|ChEBI:17295</td><td>GLN|PHE</td></tr>
<tr><td>PWY-1782</td><td>superpathway of indole-3-acetate conjugate biosynthesis</td><td>Pathways</td><td>BioCyc</td><td>0.0055</td><td>0.1289</td><td>ChEBI:18050|ChEBI:17295</td><td>GLN|PHE</td></tr>
<tr><td>map00970</td><td>Aminoacyl-tRNA biosynthesis</td><td>Pathways</td><td>KEGG</td><td>0.0163</td><td>0.1930</td><td>ChEBI:18050|ChEBI:17295</td><td>C00064|C00079</td></tr>
<tr><td>map02010</td><td>ABC transporters</td><td>Pathways</td><td>KEGG</td><td>0.0230</td><td>0.1930</td><td>ChEBI:18050|ChEBI:17295</td><td>C00064|C00079</td></tr>
<tr><td>SMP00059</td><td>Urea Cycle</td><td>SMPDB Pathway</td><td>ECMDB</td><td>0.0617</td><td>0.2702</td><td>ChEBI:17295|ChEBI:18050</td><td>ECMDB00159|ECMDB00641</td></tr>
<tr><td>SMP00059</td><td>Urea Cycle</td><td>SMPDB Pathway</td><td>ECMDB</td><td>0.9329</td><td>0.9329</td><td>ChEBI:17295|ChEBI:18050</td><td>ECMDB00159|ECMDB00641</td></tr>
<tr><td>map01100</td><td>Metabolic pathways</td><td>Pathways</td><td>KEGG</td><td>0.9138</td><td>0.9448</td><td>ChEBI:18050|ChEBI:17295</td><td>C00064|C00079</td></tr>
<tr><th colspan="8">For random forest model</th></tr>
<tr><th>ID Annotation</th><th>Annotation</th><th>Category</th><th>Database</th><th>p-value</th><th>FDR correction</th><th>Submitted IDs</th><th>Matching IDs</th></tr>
<tr><td>map01100</td><td>Metabolic pathways</td><td>Pathways</td><td>KEGG</td><td>0.8758</td><td>0.9094</td><td>ChEBI:18050|ChEBI:17295|ChEBI:17191|ChEBI:17066</td><td>C00064|C00079|C00407|C01089</td></tr>
<tr><td>SMP00059</td><td>Urea Cycle</td><td>SMPDB Pathway</td><td>ECMDB</td><td>0.0003</td><td>0.0050</td><td>ChEBI:17295|ChEBI:17191|ChEBI:18050</td><td>ECMDB00159|ECMDB00172|ECMDB00641</td></tr>
<tr><td>SMP00019</td><td>Transcription/Translation</td><td>SMPDB Pathway</td><td>HMDB</td><td>0.0009</td><td>0.0096</td><td>ChEBI:17295|ChEBI:17191|ChEBI:18050</td><td>HMDB00159|HMDB00172|HMDB00641</td></tr>
<tr><td>map00970</td><td>Aminoacyl-tRNA biosynthesis</td><td>Pathways</td><td>KEGG</td><td>0.0036</td><td>0.0820</td><td>ChEBI:18050|ChEBI:17295|ChEBI:17191</td><td>C00064|C00079|C00407</td></tr>
<tr><td>map02010</td><td>ABC transporters</td><td>Pathways</td><td>KEGG</td><td>0.0061</td><td>0.0820</td><td>ChEBI:18050|ChEBI:17295|ChEBI:17191</td><td>C00064|C00079|C00407</td></tr>
<tr><td>SMP00059</td><td>Urea Cycle</td><td>SMPDB Pathway</td><td>ECMDB</td><td>0.0163</td><td>0.1383</td><td>ChEBI:17295|ChEBI:17191|ChEBI:18050</td><td>ECMDB00159|ECMDB00172|ECMDB00641</td></tr>
<tr><td>TRNA-CHARGING-PWY</td><td>tRNA charging</td><td>Pathways</td><td>BioCyc</td><td>0.0062</td><td>0.1463</td><td>ChEBI:18050|ChEBI:17191|ChEBI:17295</td><td>GLN|ILE|PHE</td></tr>
<tr><td>SMP00059</td><td>Urea Cycle</td><td>SMPDB Pathway</td><td>ECMDB</td><td>0.9265</td><td>0.9265</td><td>ChEBI:17295|ChEBI:17191|ChEBI:18050</td><td>ECMDB00159|ECMDB00172|ECMDB00641</td></tr>
<tr><td>PWY-6220</td><td>jasmonoyl-amino acid conjugates biosynthesis I</td><td>Pathways</td><td>BioCyc</td><td>0.0029</td><td>0.1463</td><td>ChEBI:17191|ChEBI:17295</td><td>ILE|PHE</td></tr>
<tr><td>COMPLETE-ARO-PWY-1</td><td>superpathway of phenylalanine, tyrosine, and tryptophan biosynthesis</td><td>Pathways</td><td>BioCyc</td><td>0.0043</td><td>0.1463</td><td>ChEBI:18050|ChEBI:17295</td><td>GLN|PHE</td></tr>
<tr><td>PWY-6318</td><td>phenylalanine degradation IV</td><td>Pathways</td><td>BioCyc</td><td>0.0115</td><td>0.1463</td><td>ChEBI:18050|ChEBI:17295</td><td>GLN|PHE</td></tr>
<tr><td>PWY-1782</td><td>superpathway of indole-3-acetate conjugate biosynthesis</td><td>Pathways</td><td>BioCyc</td><td>0.0072</td><td>0.1463</td><td>ChEBI:18050|ChEBI:17295</td><td>GLN|PHE</td></tr>
<tr><td>PWY-6219</td><td>indole-3-acetyl-amide conjugate biosynthesis</td><td>Pathways</td><td>BioCyc</td><td>0.0054</td><td>0.1463</td><td>ChEBI:18050|ChEBI:17295</td><td>GLN|PHE</td></tr>
<tr><td>PWY-6233</td><td>jasmonoyl-amino acid conjugates biosynthesis II</td><td>Pathways</td><td>BioCyc</td><td>0.0021</td><td>0.1463</td><td>ChEBI:18050|ChEBI:17191</td><td>GLN|ILE</td></tr>
<tr><td>map01064</td><td>Biosynthesis of alkaloids derived from ornithine, lysine and nicotinic acid</td><td>Pathways</td><td>KEGG</td><td>0.0314</td><td>0.1696</td><td>ChEBI:17295|ChEBI:17191</td><td>C00079|C00407</td></tr>
<tr><td>map00966</td><td>Glucosinolate biosynthesis</td><td>Pathways</td><td>KEGG</td><td>0.0377</td><td>0.1696</td><td>ChEBI:17295|ChEBI:17191</td><td>C00079|C00407</td></tr>
<tr><td>map00960</td><td>Tropane, piperidine and pyridine alkaloid biosynthesis</td><td>Pathways</td><td>KEGG</td><td>0.0323</td><td>0.1696</td><td>ChEBI:17295|ChEBI:17191</td><td>C00079|C00407</td></tr>
<tr><td>SMP00059</td><td>Urea Cycle</td><td>SMPDB Pathway</td><td>ECMDB</td><td>0.6315</td><td>0.7157</td><td>ChEBI:17295|ChEBI:17191</td><td>ECMDB00159|ECMDB00172</td></tr>
<tr><td>map01110</td><td>Biosynthesis of secondary metabolites</td><td>Pathways</td><td>KEGG</td><td>0.9402</td><td>0.9402</td><td>ChEBI:17295|ChEBI:17191</td><td>C00079|C00407</td></tr>
</table>
